# Supplementary material for: Modeling glioblastoma heterogeneity as a dynamic network of cell states
Source: Mol Syst Biol. 2021 Sep 16;17(9):e10105. doi: 10.15252/msb.202010105 (PMC8444284; doi:10.15252/msb.202010105)
Supplement: Supplementary file 6 — Source Data for Figure 5 [file MSB-17-e10105-s004.zip › Figure5A_sourcedata/GSEA_3071/nosigdb_stateIV.GseaPreranked.1622795164626/SUVA_NPC2_PMID_31327527.html]

Details for gene set SUVA\_NPC2\_PMID\_31327527[GSEA]

|  || Dataset | state53071 |
| Phenotype | NoPhenotypeAvailable |
| Upregulated in class | na\_pos |
| GeneSet | SUVA\_NPC2\_PMID\_31327527 |
| Enrichment Score (ES) | 0.67825377 |
| Normalized Enrichment Score (NES) | 2.8251088 |
| Nominal p-value | 0.0 |
| FDR q-value | 0.0 |
| FWER p-Value | 0.0 |
Table: GSEA Results Summary

  

Fig 1: Enrichment plot: SUVA\_NPC2\_PMID\_31327527      
 Profile of the Running ES Score & Positions of GeneSet Members on the Rank Ordered List

  

| PROBE | GENE SYMBOL | GENE\_TITLE | RANK IN GENE LIST | RANK METRIC SCORE | RUNNING ES | CORE ENRICHMENT || 1 | DCX |  |  | 4 | 2.752 | 0.0882 | Yes |
| 2 | DLX5 |  |  | 17 | 2.283 | 0.1597 | Yes |
| 3 | SOX11 |  |  | 56 | 1.811 | 0.2110 | Yes |
| 4 | TAGLN3 |  |  | 71 | 1.720 | 0.2640 | Yes |
| 5 | DPYSL3 |  |  | 75 | 1.691 | 0.3181 | Yes |
| 6 | TCF4 |  |  | 96 | 1.551 | 0.3644 | Yes |
| 7 | NFIB |  |  | 117 | 1.491 | 0.4088 | Yes |
| 8 | NFIX |  |  | 162 | 1.345 | 0.4439 | Yes |
| 9 | TUBB3 |  |  | 167 | 1.330 | 0.4861 | Yes |
| 10 | PKIA |  |  | 204 | 1.253 | 0.5198 | Yes |
| 11 | KIF5C |  |  | 240 | 1.170 | 0.5510 | Yes |
| 12 | KIF5A |  |  | 358 | 0.961 | 0.5598 | Yes |
| 13 | ATP1B1 |  |  | 359 | 0.961 | 0.5908 | Yes |
| 14 | SOX4 |  |  | 377 | 0.928 | 0.6176 | Yes |
| 15 | DPYSL5 |  |  | 411 | 0.895 | 0.6402 | Yes |
| 16 | CD24 |  |  | 545 | 0.770 | 0.6399 | Yes |
| 17 | FNBP1L |  |  | 576 | 0.741 | 0.6581 | Yes |
| 18 | DDAH2 |  |  | 655 | 0.686 | 0.6655 | Yes |
| 19 | NREP |  |  | 701 | 0.660 | 0.6783 | Yes |
| 20 | STMN4 |  |  | 876 | 0.570 | 0.6636 | No |
| 21 | CEP170 |  |  | 935 | 0.548 | 0.6703 | No |
| 22 | PAK3 |  |  | 1046 | 0.506 | 0.6657 | No |
| 23 | NDRG4 |  |  | 1266 | 0.449 | 0.6386 | No |
| 24 | TUBB2A |  |  | 1373 | 0.422 | 0.6322 | No |
| 25 | DBN1 |  |  | 1846 | 0.325 | 0.5529 | No |
| 26 | MLLT11 |  |  | 1932 | 0.308 | 0.5467 | No |
| 27 | SNAP25 |  |  | 2289 | 0.257 | 0.4874 | No |
| 28 | RND3 |  |  | 4402 | -0.635 | 0.1065 | No |
| 29 | MIAT |  |  | 4622 | -0.748 | 0.0890 | No |
| 30 | LBH |  |  | 5038 | -1.168 | 0.0479 | No |
Table: GSEA details [plain text format]

  

Fig 2: SUVA\_NPC2\_PMID\_31327527: Random ES distribution      
 Gene set null distribution of ES for **SUVA\_NPC2\_PMID\_31327527**
